# Bilayer lead oxide X-ray photoconductor for lag-free operation

**DOI:** 10.1038/s41598-020-77050-w

**Published:** 2020-11-18

**Authors:** Oleksandr Grynko, Tristen Thibault, Emma Pineau, Gytis Juska, Alla Reznik

**Affiliations:** 1 Chemistry and Materials Science Program, Lakehead University, 955 Oliver Road, Thunder Bay, ON P7B 5E1 Canada; 2 Physics Department, Lakehead University, 955 Oliver Road, Thunder Bay, ON P7B 5E1 Canada; 3 Department of Solid State Electronics, Vilnius University, Saulėtekio 9 III k., 10222 Vilnius, Lithuania; 4 Thunder Bay Regional Health Research Institute, 980 Oliver Road, Thunder Bay, ON P7B 6V4 Canada

**Keywords:** Electronic properties and materials, X-rays, Semiconductors

## Abstract

Polycrystalline Lead Oxide (poly-PbO) was considered one of the most promising photoconductors for the direct conversion X-ray medical imaging detectors due to its previous success in optical imaging, i.e., as an optical target in so-called Plumbicon video pick-up tubes. However, a signal lag which accompanies X-ray excitation, makes poly-PbO inapplicable as an X-ray-to-charge transducer in real-time X-ray imaging. In contrast, the recently synthesized Amorphous Lead Oxide (a-PbO) photoconductor is essentially lag-free. Here, we report on our approach to a PbO detector where a thin layer of a-PbO is combined with a thick layer of poly-PbO for lag-free operation. In the presented a-PbO/poly-PbO bilayer structure, the poly-PbO layer serves as an X-ray-to-charge transducer while the a-PbO acts as a lag prevention layer. The hole mobility in the a-PbO/poly-PbO bilayer structure was measured by photo-Charge Extraction by Linearly Increasing Voltage technique at different temperatures and electric fields to investigate charge transport properties. It was found that the hole mobility is similar to that in a-Se—currently the only commercially viable photoconductor for the direct conversion X-ray detectors. Evaluation of the X-ray temporal performance demonstrated complete suppression of signal lag, allowing operation of the a-PbO/poly-PbO detector in real-time imaging.

## Introduction

Direct conversion detectors opened a new era in X-ray medical imaging because of the number of advantages intrinsic to the direct conversion scheme, such as spatial resolution that is limited only by the pixel dimensions and high dose efficiency even at low radiation exposure.

In the direct conversion method, incident X-rays are absorbed in a layer of photoconductor that directly generates electron–hole pairs, which are afterwards separated by an electric field to produce an electrical signal. The photogenerated electrons and holes drift towards the opposite electrodes, making it possible to create an image with a thick detector layer while maintaining high spatial resolution. The first commercial mammographic direct conversion detectors based on amorphous selenium (a-Se) photoconductor to directly convert incident X-rays to charge, which is subsequently electronically read out by a two-dimensional array of a-Si:H thin film transistors (TFTs), made a breakthrough in the breast imaging field due to the excellent detectability of small breast lesions^1^.

Currently, the only commercially feasible photoconductor for X-ray imaging is a-Se, which limits the extensive use of the direct conversion scheme. Indeed, a-Se has a low atomic number *Z*, and thus, is efficient only when low-energy X-rays are used, e.g., in mammography. To expand the benefits of direct conversion detectors over the diagnostic energy range, i.e., in radiography and fluoroscopy, a-Se must be substituted by a high-*Z* material, which can efficiently absorb higher energy X-rays.

Since X-ray imaging detectors should normally have a large active area (one cannot converge X-rays), single crystalline photoconductors are ruled out from the application in direct conversion detectors in conjunction with large area a-Si:H flat panel technology (i.e., active matrix flat panel imagers or AMFPIs). Thus, it is imperative to focus on amorphous and polycrystalline phases of high-*Z* photoconductors, that can be directly deposited on an imaging array of a large area. Potential X-ray photoconductors such as polycrystalline layers of BiI_3_^2^, PbI_2_^3,4^, HgI_2_^5–7^, ZnO^8^, CdTe^9^, Cd_1−x_Zn_x_Te^10^, and PbO^11^ have been investigated and have shown potential for use in direct conversion detectors. The X- ray-to-charge conversion rate of these materials is 3–8 times larger than that of a-Se and thus, they are capable of X-ray quantum noise limited operation at low exposures, since the X-ray quantum noise can prevail over the electronic noise. The performance of these polycrystalline photoconductors is far from ideal due to either a large dark current, incomplete charge collection, or inadequate temporal response to X-ray irradiation, appearing as a residual current after X-ray exposure is terminated, i.e., signal lag (or from a combination of these problems). In addition, the deposition of poly-CdTe and poly-Cd_1−x_Zn_x_Te layers requires a high substrate temperature (400–600 °C), which is incompatible with most of the readout electronics, or requires a hybrid deposition method, where the film is firstly deposited on the glass or alumina substrate and then coupled to the readout circuitry. The latter involves very precise alignment of the photoconductor relative to the matrix array to achieve proper pixel-to-pixel bonding that is technologically challenging, especially for high-resolution detectors.

Another promising class of materials to be used in radiation detection are perovskite semiconductors^12^; however, they are at a very early stage of the development (suffering from material instability, signal lag and, comparatively high dark current, technological difficulties in manufacturing a thick and uniform layer over a large area) and are not mature enough to be considered as a practical solution to improve radiation medical imaging.

It should be mentioned that among the potential X-ray-to-charge transducers listed above, polycrystalline Lead Oxide (poly-PbO) is especially promising because, similarly to a-Se, it has a long history of commercial utilization in optical imaging. Previously, thin layers of poly-PbO were used in “Plumbicon” video pick-up tubes that were widely used for broadcast, fluoroscopy and digital subtraction angiography in conjunction with image intensifiers. However, the first prototype of poly-PbO based X-ray detector with the radiography-thick photoconductor film was unsuccessful. The photoconductor exhibited signal lag^13^ that did not permit the use of PbO detectors in real time fluoroscopic imaging, which is the most clinically demanding application^14^.

Recently, we reported on the development of a new polymorphic form of the PbO material, namely amorphous Lead Oxide (a-PbO), which had not been previously synthesized^15^. In contrast to its polycrystalline counterpart, a-PbO is dense, capable of withstanding higher electric fields with lower dark current and exhibits no signal lag^16^. In this work, we use a-PbO in a bilayer PbO structure, where a thick layer of poly-PbO serves as a recording and charge transport layer while a thin layer of a-PbO acts as a lag-preventing layer.

The use of multilayer photoconductive structures is a standard solution in direct conversion detectors. Indeed, practical a-Se direct conversion detectors utilize a multilayered structure, consisting of a thick photoconducting layer of stabilized a-Se, sandwiched between one or two adjacent blocking layers needed to maintain an acceptable level of dark current.

In the majority of those detectors, two blocking layers are identified as *n*-like (hole blocking) and *p*-like (electron blocking). Examples of *n*-like layers are a-Se alloyed with a small percent of As and doped with alkali metal (usually Na or LiF)^17–23^, cold-deposited a-Se^19,20^, Sb_2_S_3_^24^ and CeO_2_^23,25^. Among *p*-like layers are As_2_Se_3_^17–20,24^, a-Se doped with Cl^22^, and Sb_2_S_3_^24–26^. Additional layers may be used, such as Arsenic-rich a-Se layers to retard crystallite formation at the interface^24,26,27^; and protective overcoating layer such as nigrosine^28^, Parylene^29^, epoxy resin^26^. As it is seen, the manufacturing of an a-Se based detector requires multistage doping and alloying with different elements, normally performed in one deposition cycle without breaking the vacuum. This process is technically challenging and requires precise control to avoid cross-contamination, leading to high deposition cost^30^.

Our approach to combine two allotropic forms of the same material, namely, PbO, in a blocking structure reduces technical complexity, in comparison with a-Se technology. At the same time, it will allow to combine excellent (low) ionization energy inherent to PbO, adequate hole mobilities and low dark current at practical electric fields. This holds a potential to make the direct conversion system simpler and hence, more economical to manufacture. Although we do not claim that the developed technology can be immediately used in commercial products, we show that it allows for lag-free real-time imaging in the diagnostic energy range. We also report on the direct measurements of hole mobilities and demonstrate, for the first time, the application of the bilayer PbO structure as a direct conversion X-ray detector.

## Results

### Experimental sample

**Figure 1:**
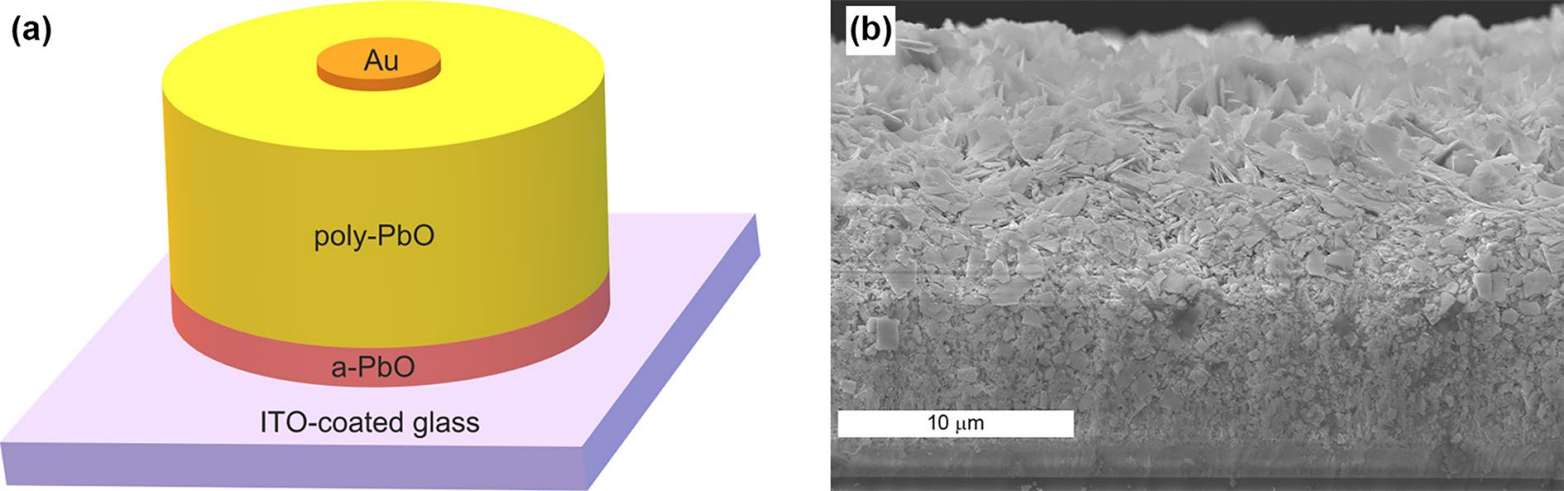
Schematic diagram (**a**) and cross-sectional SEM image (**b**) of a bilayer a-PbO/poly-PbO structure.

A schematic presentation of a bilayer Lead Oxide structure is shown in Fig. 1a. The bottom 2 μm lag-preventing a-PbO layer was deposited on an ITO-coated glass substrate (bottom biased electrode) by an ion-assisted thermal evaporation technique^31^. Subsequently, the a-PbO layer was covered by a 12 μm layer of poly-PbO (recording and charge transport layer) using conventional thermal evaporation^31^. Although this sequential deposition of amorphous and polycrystalline layers was performed in a single deposition cycle (without breaking the vacuum), it should be noted that it can be done in separate evaporators, giving essentially identical results. Finally, a 200 nm thick Au contact was sputtered atop a-PbO/poly-PbO structure by magnetron sputtering to serve as the top readout electrode.

The SEM image of the cross-section of the sample is shown in Fig. 1b. As can be seen, the thin and dense layer of amorphous material in the lower part of the structure transforms into a less dense, inhomogeneous disordered layer of polycrystalline material. It should be noted that the structure of the poly-PbO layer in the presented bilayer structure is slightly different from the previously reported single poly-PbO layer structure. Indeed, a single poly-PbO film is essentially a porous network of individual platelets oriented mainly in the growth direction^31^, whereas the layer in the current sample consists of overlapping flakes.

### Mobility measurements

Hole mobility was measured by the photo-Charge Extraction by Linearly Increasing Voltage (CELIV) technique in a range of electric fields (0.03–0.22 V/μm) and temperatures (260–350 K). The choice of CELIV was determined by its proven efficacy in the evaluation of field and temperature dependencies of mobilities in single poly-PbO layers, characterized by dispersive carrier transport with mobilities which decrease in the course of time. This feature makes a conventional Time-of-Flight (TOF) technique ineffective for transport characterization. In contrast, the theory of CELIV was recently extended to dispersive transport regime^32^ making it a technique of choice as an alternative to TOF for disordered materials with dispersive transport.

**Figure 2:**
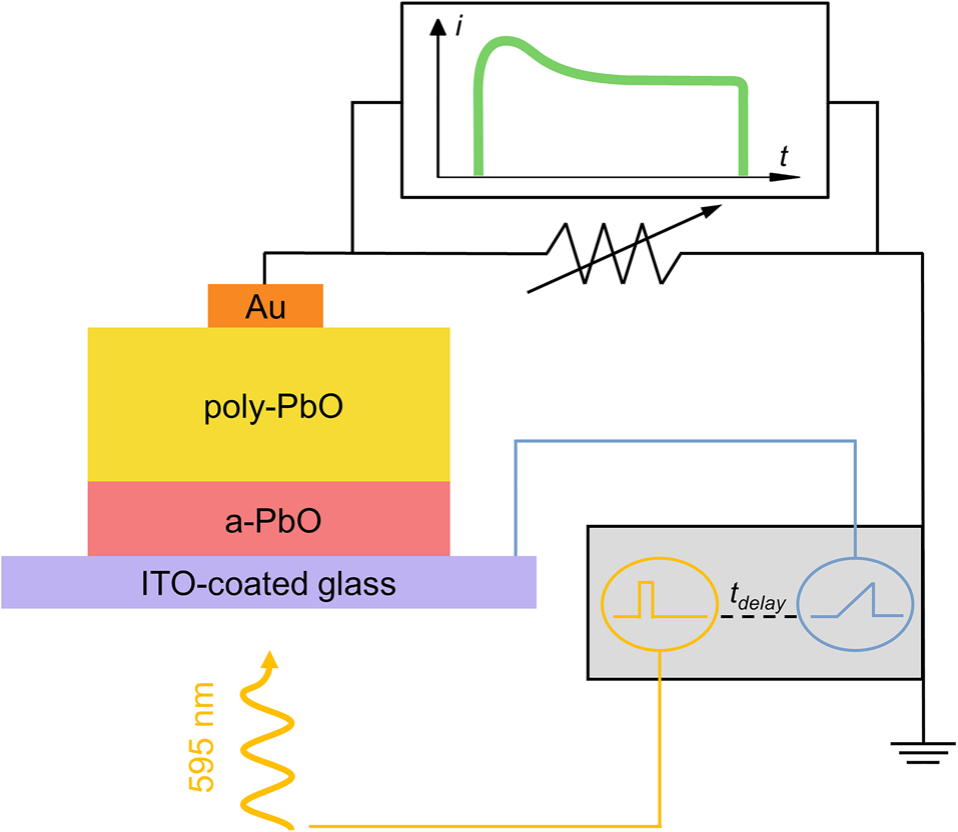
Schematic diagram of photo-CELIV experimental apparatus.

In photo-CELIV, a light pulse is used to generate charge carriers in the volume of an unbiased sample. The wavelength of optical excitation (595 nm) was chosen to provide a uniform bulk absorption so that electron–hole pairs are generated in the volume of the layer. Subsequently, a linearly increasing voltage is applied to the sample to extract the photo-generated carriers. As the applied voltage increases, so does the photocurrent since charge carriers are extracted faster. At the end of the extraction, when most carriers have been collected, the photocurrent decays (Fig. 2).

The carrier mobility is derived from the measured time needed to reach the peak of the photocurrent. For disordered materials with dispersive transport, the mobility is calculated using an equation

1 $$\mu =\frac{\alpha \left(\alpha +1\right)}{2\alpha +1}\frac{{d}^{2}}{{t}_{peak }^{2}A},$$

where *d*—detector’s thickness, *t*_*peak*_—time to the peak of the photocurrent, *A*—slope of the voltage ramp, *α*—dispersion parameter derived from the relationship ^32^

2 $${t}_{peak} \sim {A}^{- \frac{1}{1+\alpha }}.$$

**Figure 3:**
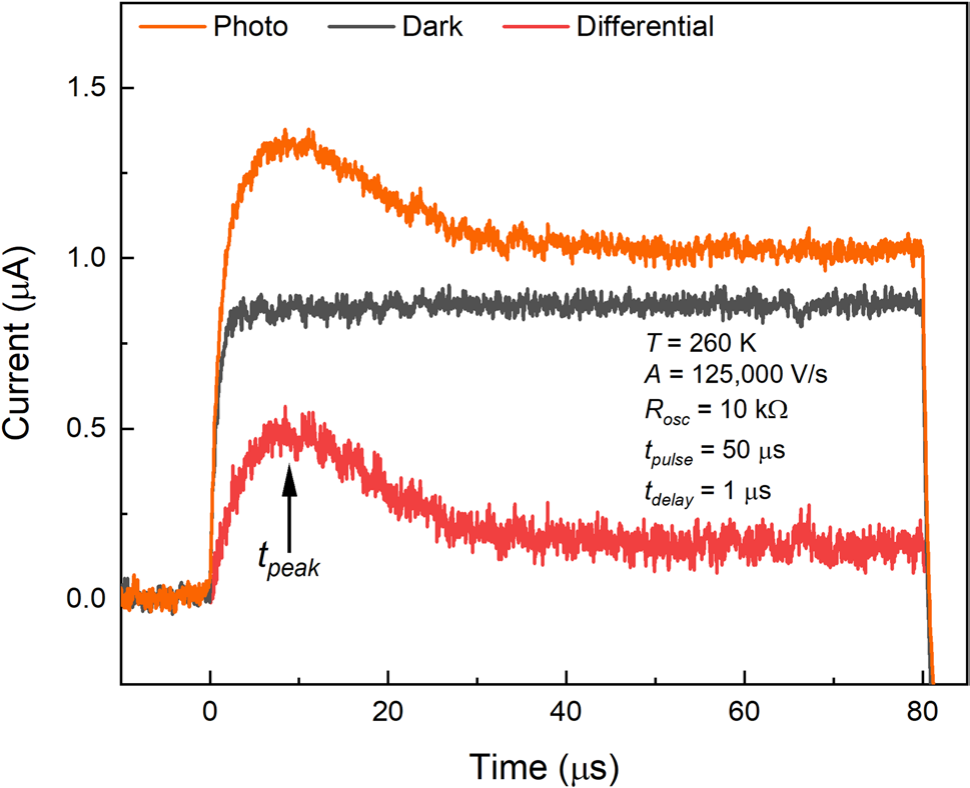
A typical photo-CELIV, dark-CELIV current transients, and differential curve. The peak time is measured from the differential signal, exhibiting an apparent photo-peak.

A typical CELIV response at given experimental conditions is shown in Fig. 3, where *T*—temperature, *R*_*osc*_—input resistance of the oscilloscope, *t*_*pulse*_—duration of a light pulse, and *t*_*delay*_—time delay between the light and voltage pulses. The dark-response, which is a capacitive component of the current, was subtracted from the photo-response, and the peak time was measured from the differential photocurrent transient.

To satisfy the requirements of the CELIV model and to prevent the accumulation of space charge^33^, the magnitude and duration of the light pulse were adjusted to make the amplitude of the photocurrent (differential signal) smaller than the capacitive (dark) signal; and the input resistance of the oscilloscope was adjusted to minimize the RC-component of the signal. Also, since it has been previously shown that holes are much faster carriers in poly-PbO^32,34^, the experimental conditions were tuned (i.e., a voltage pulse of low amplitude and short duration was used throughout the experiments) to make the photocurrent caused by the drift of holes only, so that slower electrons do not contribute to the recorded photocurrent.

**Figure 4:**
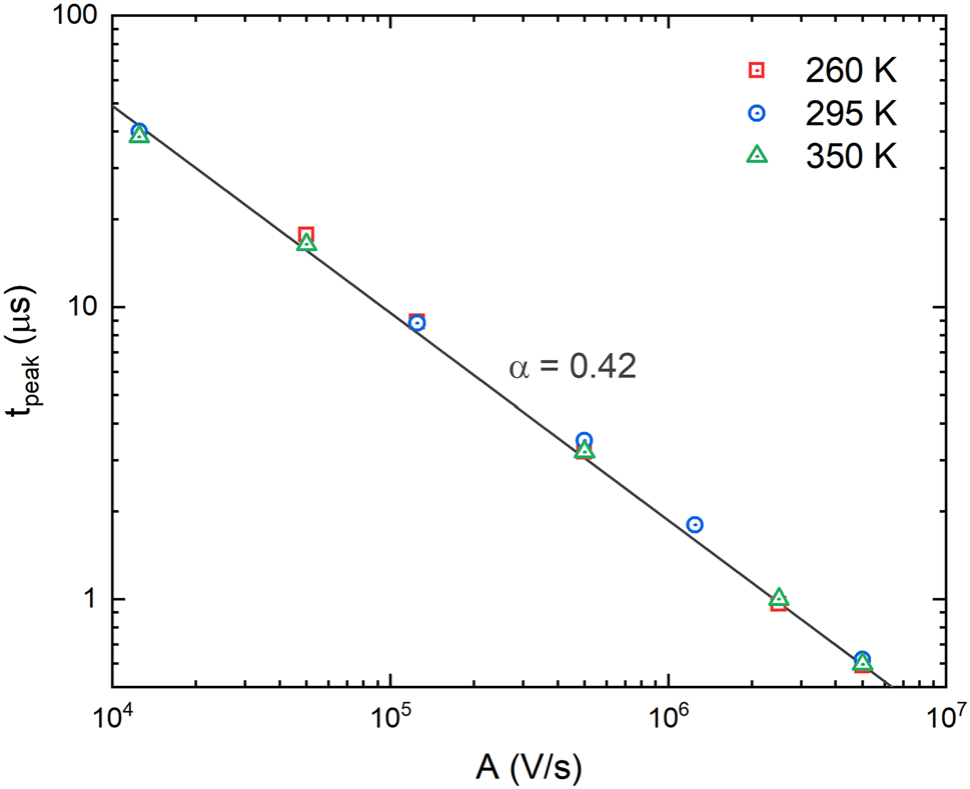
Time to peak dependence on voltage ramp at different temperatures. The dispersion parameter derived from a slope of a log–log fit line is independent of temperature.

Figure 4 shows experimentally measured time to peak dependence on the voltage ramp at different temperatures. A slope of a log–log fit curve yields a value of the dispersion parameter *α* = 0.42, from Eq. (2). The dispersion parameter is temperature independent, similarly to what has been found in single poly-PbO layers^32^; however, its value is twice larger than that in single poly-PbO.

**Figure 5:**
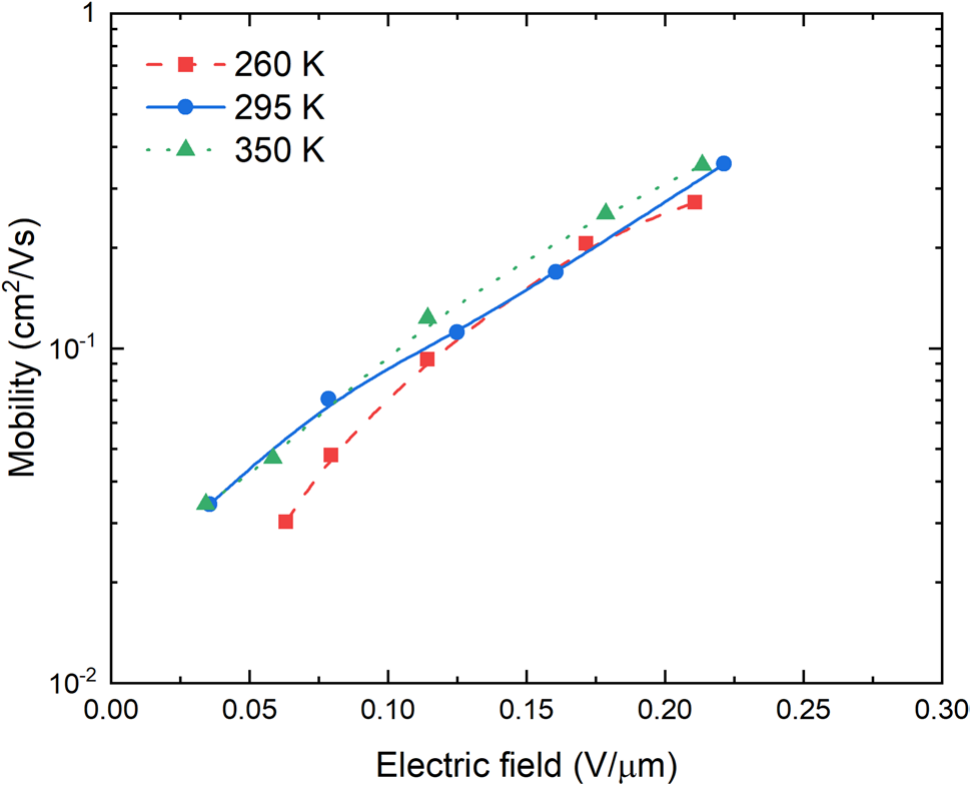
Hole mobility dependence on electric field at different temperatures. The mobility is independent of temperature but have a strong dependence on electric field.

The mobility of holes *μ*(*F*) was calculated using Eq. (1) for electric field at the time of maximum extraction $\left(F=\frac{A\cdot {t}_{peak}}{d}\right)$ at different voltage ramps *A* and temperatures (Fig. 5). Hole mobility is strongly dependent on the electric field and reaches a value of 0.35 cm^2^/Vs at 0.22 V/μm. To compare, the mobility of holes in a-Se is 0.15 cm^2^/Vs at 1 V/μm at room temperature^35^. The weak dependence of mobility on temperature and the strong dependence on applied field indicates a dispersive transport regime mainly governed by spatial inhomogeneity^32^.

### Temporal performance evaluation

**Figure 6:**
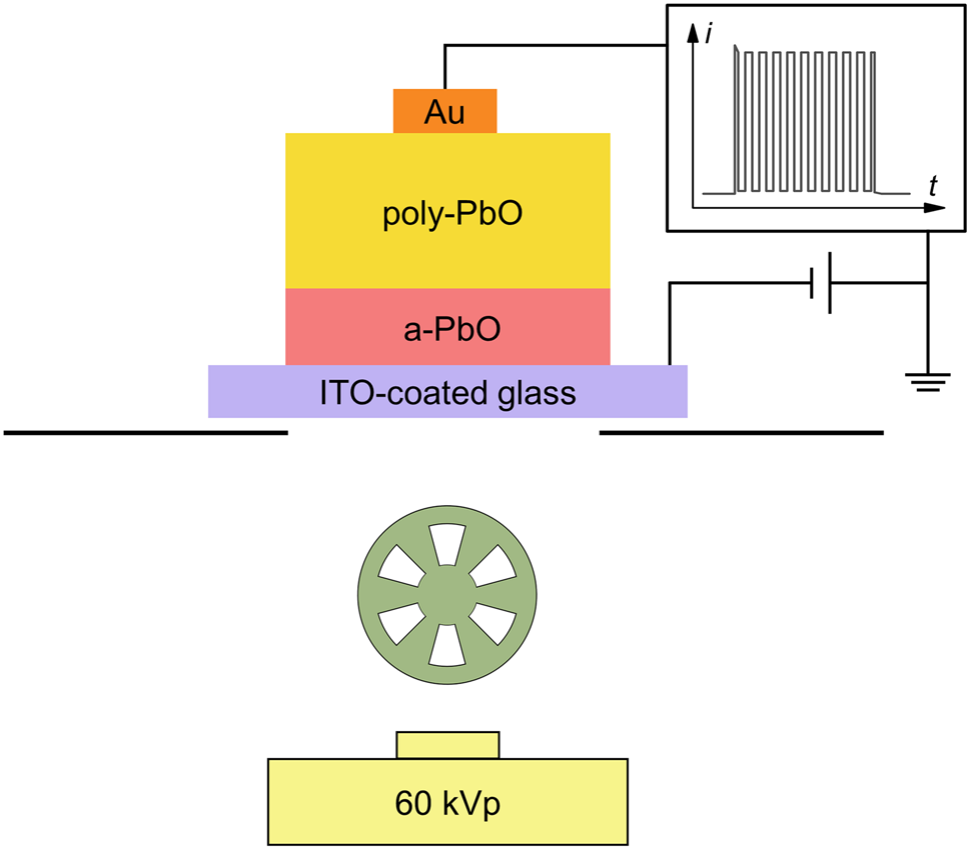
Schematic diagram of XPM setup in a fluoroscopic mode.

The temporal performance was examined by the X-ray induced Photocurrent Method (XPM) in a pulsed mode. The photocurrent in a bilayer PbO structure was measured at an electric field of 10 V/μm (negative bias on ITO) under 1 s long X-ray exposure, modulated using a rotating chopper at various frequencies in the range of 10–60 Hz and 50% duty cycle (Fig. 6). In this test, image lag would reveal itself as an increase in the signal in each frame^36^.

**Figure 7:**
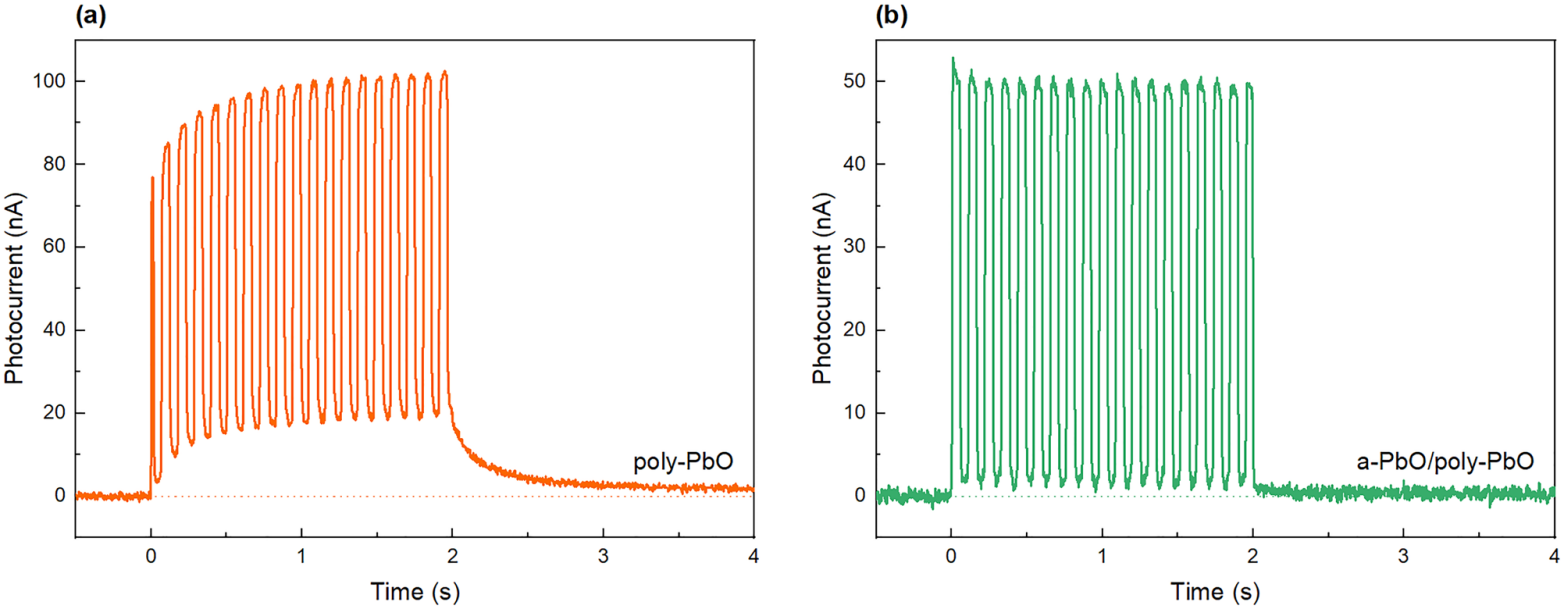
Comparison of X-ray response to pulsed irradiation of (**a**) poly-PbO and (**b**) bilayer a-PbO/poly-PbO detectors. Poly-PbO exhibits signal build-up and prolonged residual signal after the termination of X-ray pulse, while bilayer PbO detector shows perfect temporal response. It should be noted that the poly-PbO sample used in this experiment is thicker than a-PbO/poly-PbO, thus it yields a higher amplitude of X-ray response.

To demonstrate this, Fig. 7 compares outputs of a bilayer PbO structure and a single poly-PbO layer biased at 5 V/μm and irradiated with X-rays pulsed at a frequency of 10 frames per second (fps). The exposure was 2 s long, during which both samples were exposed to 20 X-ray pulses (100 ms alternating intervals with open and closed chopper). When irradiated with pulsed X-rays, a rise of the photocurrent level with each subsequent frame is evident in the single poly-PbO layer. In addition, there is a well-pronounced residual signal, that decays to zero in approximately 2 s after the termination of X-ray exposure. In contrast, a bilayer a-PbO/poly-PbO structure shows a stable photocurrent value and no signal lag.

**Figure 8:**
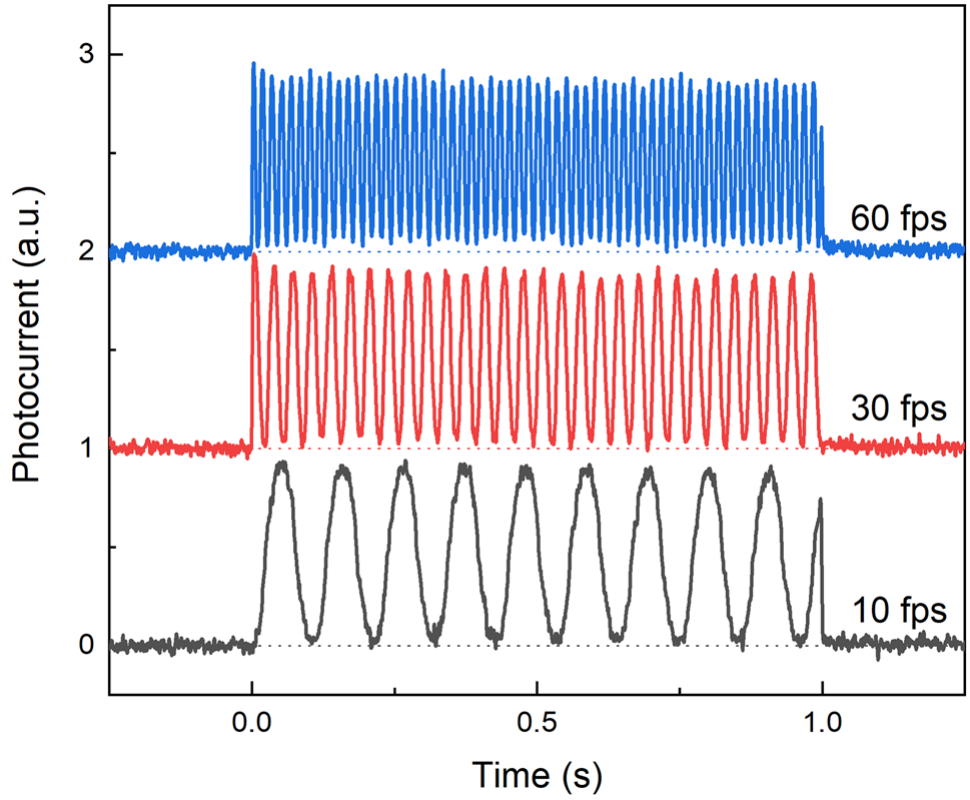
A bilayer detector’s response to X-ray irradiation pulsed at different frequencies. Each waveform was normalized and shifted for better visualization. The first and the last frames are affected by asynchronization between the chopper and X-ray pulse, and nonuniformity of exposure at the beginning of the pulse.

The temporal performance of the bilayer detector shows a nearly constant amplitude of the signal in each frame even at a high readout rate (Fig. 8). A uniform amplitude during the X-ray exposure indicates no build-up of the injection current, allowing maintenance of constant dark current. After the termination of the X-ray pulse, the photocurrent drops rapidly to zero level with no residual signal, proving lag-free operation.

Frequency of 30 fps used in this experiment corresponds to readout rate conventionally used in a real-time fluoroscopy imaging. A reduced frame rate can be used to increase the signal-to-noise ratio (SNR) of the detector and to moderate patient dose. A higher readout rate might be necessary when one must image a rapidly changing process, such as heart beating (cardiac imaging and coronary catheterization)^37^.

## Discussion

The performance of a-PbO/poly-PbO bilayer structure has been investigated in terms of whether combining these PbO polymorphs into a multilayer structure allows to consolidate the adequate hole mobility inherent in poly-PbO with the excellent X-ray temporal response inherent in a-PbO layers. CELIV measurements confirmed that hole transport in the bilayer structure is dispersive and qualitatively similar to that in single layers of poly-PbO^32^. Weak temperature dependency of mobility *μ* and dispersion parameter *α*, and strong field dependence of *μ* indicates that dispersion is governed by spatial disorder rather than energy disorder. Mobility of holes reaches value of 0.35 cm^2^/Vs at relatively low field 0.22 V/μm (the upper value of the applied electric field was limited by the bandwidth of the function generator). The fact that mobility increases rapidly with an increase in electric field is very desirable since it provides a tool to improve the carrier schubweg i.e. the average distance drifted before a carrier is lost to traps (schubweg is a product of the carrier drift mobility *µ*, the lifetime *τ* and field *F*, i.e. *µτF*) and to avoid depth dependent charge collection. Both the dispersion parameter and hole mobility obtained for a bilayer a-PbO/poly-PbO structure are somewhat higher than those for poly-PbO. This can be attributed to the differences in structural heterogeneity between a single poly-PbO and that grown on a-PbO: depositing a poly-PbO layer on a-PbO sublayer makes hole transport less dispersive and increases hole mobility.

Evaluation of the temporal response of the bilayer structure with the pulsed X-ray radiation did not reveal signal lag—a major obstacle for application of poly-PbO layers in the direct conversion X-ray detectors. Indeed, the presence of signal lag and subsequently, image lag, does not permit to use poly-PbO detectors in the most demanding clinical application that is real time fluoroscopic imaging. Dynamic readout, as that used in real time applications, requires very little to no lag at the end of each frame, otherwise previous images will be superimposed with the subsequent ones, thus resulting in a misleading view. The elimination of signal lag can be explained by decreasing charge trapping at a-PbO/poly-PbO interface and suppressing X-ray triggered injection caused by local enhancement of the electric field at the interface between the electrode and photoconductor^13^. In a bilayer configuration, a lag-preventing layer of a-PbO separates poly-PbO from the electrode, preventing charge injection and eliminating image lag.

Despite this, the dark current of a bilayer structure is still higher than the recommended value for digital flat panel detectors (~ 10 pA/mm^2^)^38^. Further decrease in the dark current may be achieved by using a thin blocking layer between the electrodes and PbO bilayer. One of the most suitable candidates is polyimide (PI). It is a well-developed material that was already successfully used with a-Se photoconductors^36^. Careful optimization of the film’s thickness allows the tuning of its resistivity to prevent significant voltage drop across the blocking layer as well as redistribution of the electric field inside the structure^39^. It should be noted that polyimide film can be easily applied by a conventional spin coating technique, in contrast to complicated process of growing of the doped layers mentioned earlier. The development of the PI blocking layer to be used in a-PbO/poly-PbO-based X-ray detectors is left for future work.

Overall, our results demonstrate that combining an a-PbO lag-preventing layer with a poly-PbO X-ray-receiving layer in a bilayer structure allows to obtain lag-free operation while preserving adequate hole mobilities (relative to other disordered photoconductors) needed for adequate schubweg and high efficiency in collecting X-ray generated charge.

## Methods

### Sample preparation

The bilayer PbO sample was thermally evaporated on Indium Tin Oxide (ITO) coated glass substrate. To form a-PbO layer, a high purity (5 N) yellow PbO powder was evaporated at the temperature ~ 1000 °C in atmosphere of ionized oxygen at the pressure ~ 0.1 Pa. The growing layer was continuously bombarded by Oxygen ions with energy ~ 70 eV. The poly-PbO layer was formed from the same powder and at the similar temperatures, but in contrast to a-PbO, evaporation took place in atmosphere of molecular oxygen at the pressure ~ 0.3 Pa. The substrate temperature was kept below 150 °C in both processes. A top gold contact was sputtered ex situ in a dedicated chamber. Since poly-PbO is known to degrade under ambient conditions by forming a hydro-cerussite compound^14^, all measurements were performed in the protective atmosphere of dry Nitrogen.

### CELIV apparatus

A sample was placed in a heating–cooling stage Linkam LTS350 that enables a precise temperature control in a wide range of temperatures. A 2-channel function generator Tektronix AFG 3022C was used to apply a linearly increasing voltage pulse on ITO and trigger a light pulse from the ITO side. The current transients were readout from Au electrode on the oscilloscope Tektronix TDS 2024C with adjustable input resistance. For an excitation LED light pulse with a radiant power ~ 100 μW and a wavelength of 595 nm, the attenuation depts in Lead Oxide is 90 μm^40^, which is much larger than the detector’s thickness of 14 μm. This provides a homogeneous generation of charge carriers through the bulk of the film. The range of electric fields used in these measurements was limited by the bandwidth of the function generator.

### XPM apparatus

The sample was placed in the shielded Aluminum box with a 2-mm thick lead collimator. A negative DC bias was applied to the ITO from a high voltage power supply (Stanford Research Systems PS350), and the photocurrent was readout from the Au electrode on oscilloscope Tektronix TDS 2024C with input resistance of 1 MΩ. The X-ray tube (Dunlee PX1412CS, insert DU-304) with a Tungsten target and a 2-mm Al filter was used to generate 60 kVp X-ray pulse (tube current was 200 mA). A chopper controller (Stanford Research Systems SR540) drives a 2-mm thick Copper chopper to modulate an X-ray pulse at different frequencies and 50/50 duty cycle that was used to simulate a pulsed irradiation mode.

## Data Availability

The datasets generated and analysed during the current study are available from the corresponding author on reasonable request.
